# Negative regulation of the interferon response by an interferon-induced long non-coding RNA

**DOI:** 10.1093/nar/gku713

**Published:** 2014-08-13

**Authors:** Hiroto Kambara, Farshad Niazi, Lenche Kostadinova, Dilip K. Moonka, Christopher T. Siegel, Anthony B. Post, Elena Carnero, Marina Barriocanal, Puri Fortes, Donald D. Anthony, Saba Valadkhan

**Affiliations:** 1 Department of Biochemistry, School of Medicine, Case Western Reserve University, Cleveland, OH 44106, USA; 2 Divisions of Infectious and Rheumatic Diseases, Department of Medicine, School of Medicine, Case Western Reserve University, Cleveland, OH 44106, USA; 3 Division of Gastroenterology, Henry Ford Health System, Detroit, MI 48202, USA; 4 Department of Surgery, University Hospitals Case Medical Center, Cleveland, OH 44106, USA; 5 Department of Gastroenterology, University Hospitals Case Medical Center, Cleveland, OH 44106, USA; 6 Department of Hepatology and Gene Therapy, Center for Applied Medical Research (CIMA), University of Navarra, Pamplona, Spain

## Abstract

Long non-coding RNAs (lncRNAs) play critical roles in diverse cellular processes; however, their involvement in many critical aspects of the immune response including the interferon (IFN) response remains poorly understood. To address this gap, we compared the global gene expression pattern of primary human hepatocytes before and at three time points after treatment with IFN-α. Among ∼200 IFN-induced lncRNAs, one transcript showed ∼100-fold induction. This RNA, which we named lncRNA-CMPK2, was a spliced, polyadenylated nuclear transcript that was induced by IFN in diverse cell types from human and mouse. Similar to protein-coding IFN-stimulated genes (ISGs), its induction was dependent on JAK-STAT signaling. Intriguingly, knockdown of lncRNA-CMPK2 resulted in a marked reduction in HCV replication in IFN-stimulated hepatocytes, suggesting that it could affect the antiviral role of IFN. We could show that lncRNA-CMPK2 knockdown resulted in upregulation of several protein-coding antiviral ISGs. The observed upregulation was caused by an increase in both basal and IFN-stimulated transcription, consistent with loss of transcriptional inhibition in knockdown cells. These results indicate that the IFN response involves a lncRNA-mediated negative regulatory mechanism. lncRNA-CMPK2 was strongly upregulated in a subset of HCV-infected human livers, suggesting a role in modulation of the IFN response *in vivo*.

## INTRODUCTION

Recent global gene expression analyses have revealed that, while protein-coding sequences occupy <2% of the genome in mammalians, a much larger fraction of the genome is transcribed into long non-coding transcripts (1,2). These transcripts, the long non-coding RNAs (lncRNAs), constitute a novel layer of regulatory factors with important roles in almost every aspect of cellular function (3–9). The expression of many lncRNAs is tightly regulated by various cellular signals, including stress signaling (10–14), tissue or differentiation-specific signals (15–18) and hormones (19–21). Emerging evidence points to the involvement of lncRNAs in many aspects of the immune response, including several pathways related to innate immunity (22–24). Carpenter *et al*. (25) have shown that the newly identified lncRNA-Cox2 was upregulated upon stimulation of Toll-like receptors and played a role in regulating the inflammatory response. In addition, lncRNA THRIL and a pseudogene-derived lncRNA named Lethe were shown to be induced in response to tumor necrosis factor alpha (TNF-α), creating a negative feedback loop in the nuclear factor-κB pathway (26,27). Most recently, Imamura *et al.* reported that lncRNA NEAT1 regulates interleukin-8 expression through transcription factor SFPQ (28). Despite these exciting discoveries, the role of lncRNAs in key aspects of the immune response including the nearly ubiquitous and functionally crucial interferon (IFN) response remains largely unstudied.

The IFN response is a central component of the innate immune system and all three classes of mammalian IFNs (types I, II and III) have been shown to possess antiviral activity (29–31). Binding of IFNs to their receptors triggers the JAK-STAT signaling pathway, which in turn leads to transcriptional upregulation of hundreds of IFN-stimulated genes (ISGs) (32–34). IFNs are especially effective against viral infections, and their potent antiviral action against hepatitis C virus (HCV) has made HCV-based *in vitro* models uniquely suitable for the study of antiviral activity of the IFNs (35–39). Using these model systems, recent studies have revealed that several ISGs have significant antiviral activity against HCV in either additive or synergistic ways (40–42). However, current studies of ISGs suggest that their combined antiviral activity is not sufficient to account for the entirety of the antiviral action of the IFN response, raising the possibility that additional, novel ISGs may possess significant antiviral activities. While many protein-coding ISGs are actively studied for their antiviral activity (43–46), the contribution of long non-coding transcripts to the IFN response has not been investigated. A recent high-throughput analysis in primary mouse lung epithelial cells by Peng *et al.* (47) has shown that a number of host lncRNAs were upregulated in response to viral infection and IFN-β, although whether the observed upregulation has functional significance remains to be determined. However, study of the role of small non-coding RNAs has shown that expression of cellular microRNAs is modulated by IFN to combat viral infection (48), raising the intriguing possibility that novel functional lncRNAs may be similarly regulated by IFNs and in turn contribute to the antiviral activity of the IFN response. As many lncRNAs show rapid evolutionary divergence even between closely related species, the pattern of induction and function of lncRNAs may be highly species-specific (49–51) underscoring the need for analysis of IFN-induced lncRNAs in human cells.

Here we describe the JAK-STAT signaling pathway-dependent induction of a nuclear-localized multiexonic, alternatively spliced lncRNA in response to IFN in primary human hepatocytes and other cells, which appears to regulate other IFN-responsive genes and affect HCV replication. Our results indicate that the IFN-induced lncRNA-CMPK2 acts as a negative regulator of the IFN response, at least partially through suppression of transcription of a subset of antiviral ISGs. Further, the level of lncRNA-CMPK2 was increased in liver samples from patients chronically infected with HCV, raising the possibility that it may play a similar regulatory role *in vivo*.

## MATERIALS AND METHODS

### Plasmids and reagents

Human recombinant IFN-α was purchased from PBL Biomedical laboratory. The IFN was diluted with phosphate buffered saline (PBS) and stocked at −80°C. JAK inhibitor ruxolitinib was purchased from Fisher Scientific. Actinomycin D was purchased from Life Technologies. Lentiviral shRNA control plasmid (Sigma clone SHC002) and STAT2 shRNA plasmids (Sigma clone ID #1: TRCN0000364400 and #2: TRCN0000007460) were purchased from Sigma. Lentiviral shRNA constructs for lncRNA-CMPK2 (Target sequence #1: GGAGTGCAGTGGTGCGATA and #2: CGATGCATGGGAAGACTAA) were generated by inserting polymerase chain reaction (PCR) products into pLKO.1 vector backbone (Sigma), which is also the backbone used in control, non-targeting shRNA plasmid. The following antibodies were used: anti-beta-actin (Sigma, A2228), anti-STAT2 (Pierce, PA5-11629), Goat anti-Mouse IgG (Invitrogen, G-21040) and Goat anti-Rabbit IgG (Invitrogen, G-21234). HCV constructs were kindly provided by Dr Takaji Wakita (National Institute of Infectious Diseases, Tokyo).

### Cell culture

All cells were cultured at 37°C in a humidified atmosphere at 5% CO_2_. Plateable cryopreserved human hepatocytes from five individual donors were purchased from Celsis In Vitro Technologies. All donors were negative for human immunodeficiency virus (HIV), HCV, hepatitis B virus (HBV) and cytomegalovirus (CMV) (see Supplementary Figure S1A for additional donor information). Primary hepatocytes were counted using Trypan blue exclusion method to determine cell viability. The hepatocyte cultures that showed >80% viability were used in this study. The cells were seeded onto collagen-coated plates and cultured with InVitroGRO hepatocyte media complemented with Torpedo Antibiotic Mix (Celsis). Human hepatocellular carcinoma Huh7.5 cell line (kindly provided by Dr Charles Rice), HeLa, H1975 (kindly provided by Dr Afshin Dowlati) and 293T cell lines were maintained in Dulbecco's modified Eagle's medium (Life Technologies Corporation) supplemented with 10% fetal bovine serum (FBS) and 1% penicillin/streptomycin. Jurkat and THP1 cell lines (kindly provided by Dr Jonathan Karn) were maintained in RPMI 1640 (Life Technologies Corporation) supplemented with 10% FBS and 1% penicillin/streptomycin. Primary human keratinocytes (kindly provided by Mary Consolo at the Skin Disease Research Center's Cell Culture and Molecular Technology Core at Case Western Reserve University) were maintained in keratinocyte-SFM media (Gibco).

Cells were plated for experiments ensuring that cellular density at all plates were equal, and that the level of harvested total cellular RNA was closely similar between the control and test cells. Experiments were repeated a minimum of three times, with at least two biological repeats. In experiments with ruxolitinib, Huh7.5 cells were treated with the JAK inhibitor ruxolitinib (0.8 μM) for 1 h, with or without a subsequent treatment with IFN-α (100 units/ml) for 8 h followed by harvest of cellular RNA. To generate STAT2-knockdown cells, cells were stably transfected with vectors containing one of the two shRNA constructs against STAT2 (see above) and were subsequently treated with IFN-α (100 units/ml) or vehicle for 8 h, followed by harvest of cellular RNA. For actinomycin D blockage of transcription, knockdown or control cells were treated with IFN-α. After 9 h, the medium was replaced with one containing 3 μg/ml of actinomycin D and the cells were collected for RNA extraction at indicated time points followed by detection of the level of desired genes by reverse transcription-quantitative PCR (RT-qPCR). In experiments that involved testing the induction of lncRNA-CMPK2 by cytokines, IFN-γ and TNF-α were added to the cell culture media at 100 and 20 ng/ml concentrations, respectively.

### High-throughput sequencing

Total cellular RNA was extracted from primary hepatocytes using TRIzol Reagent (Invitrogen) followed by DNase I treatment (Affymetrix), phenol:chloroform extraction and ethanol precipitation. The RNA pellets were dissolved with RNase free water and treated by Ribo-Zero rRNA Removal kit (Epicenter) to remove ribosomal RNAs. RNA-seq libraries were made with Scriptseq v2 (Epicenter) or TruSeq stranded total RNA (illumina) according to the manufacturer's instructions. Sequencing was performed on an Illumina HiSeq 2500 instrument at a depth of ∼70 million paired-end, 100 bp long, strand-specific reads per sample. Two independent RNA-seq experiments were performed several months apart using different library preparation kits (see above) to ensure the reproducibility of the data. RNA-seq reads were aligned to Hg19 using Tophat 2 (52) with transcriptome annotations for protein-coding and non-coding RNAs obtained from both UCSC Genome Browser website (53) and the GENCODE Project (54), release 17. Mapped sequence reads were visualized and analyzed using the Integrative Genomics Viewer (www.broadinstitute.org/igv) (55,56) and SeqMonk Mapped Sequence Analysis Tool (www.bioinformatics.bbsrc.ac.uk/projects/seqmonk). In parallel, the data were analyzed using the Cufflinks suite as described (57). RNAs that reproducibly showed over 4-fold induction compared to control in a statistically significant manner (*P*-value < 0.05) were selected for further analysis (Supplementary Table S2).

### Characterization of lncRNA-CMPK2 sequence

Rapid Amplification of cDNA Ends (RACE) was performed using SMARTer RACE cDNA Amplification Kit (Clontech) according to manufacturer's instructions on total cellular RNA. We used RNA obtained from both control and IFN-stimulated cells to identify the relevant, IFN-induced species. To identify any potential isoforms, RT-PCR reactions were performed using primers that targeted the 5′ and 3′ ends of the RNA (Supplementary Table S1). The amplified species were resolved on a gel and individually purified, followed by sequencing to define the sequences present in each species and verify the exon–exon junctions. Open reading frames (ORFs) were analyzed using ORF finder (www.ncbi.nlm.nih.gov/gorf/orfig.cgi). Sequence alignments were performed using GENETYX Mac 15.0.1. RNA-seq data from this study will be deposited at the NCBI Gene Expression Omnibus or SRA (accession code will be provided).

### Subcellular fractionation

Isolation of nuclear and cytoplasmic fractions was performed as previously described (58). Briefly, Huh7.5 cells were treated with IFN-α at 500 units/ml and incubated for 12 h along with vehicle-treated control. Next they were lysed with a hypotonic buffer (50 mM Tris–HCl pH 7.4, 1.5 mM MgCl_2_, 1 mM Dithiothreitol (DTT), 10 mM KCl) containing protease inhibitor cocktail (Roche) and incubated for 20 min on ice. Subsequently the cells were homogenized with 30 strokes of Dounce homogenizer followed by addition of Triton-X (final concentration 0.1%) and centrifuged at 1200 rpm for 10 min. The pellet, corresponding to the nuclear fraction, was washed twice with hypotonic buffer. The supernatant, corresponding to the cytoplasmic fraction, was further centrifuged at 13 000 rpm at 4°C for 30 min. RNA content of nuclear and cytoplasmic fractions was extracted with TRIzol Reagent.

### RT-PCR

cDNA was generated with PrimeScript RT-PCR Kit (TAKARA Bio) using both oligo(dT) and random hexamers. For strand-specific RT-qPCR, specific reverse primers (Supplementary Table S1) were used in the RT reaction using MMLV reverse transcriptase (Invitrogen). The resulting cDNA was used in qPCR reactions with Biorad SYBR Green Kit (Biorad) on a Mastercycler Realplex2 system (Eppendorf). The results were normalized to β-actin or glyceraldehyde 3-phosphate dehydrogenase (GAPDH) according to the manufacturer's protocol. Samples showing over one Ct value difference in the level of housekeeping genes compared to controls were not used in analysis to ensure that any observed difference in the expression level of target genes was not an artefact of the difference in the level of input. The results of all biological replicates (minimum of two) and technical replicates (minimum of two) were used to derive the final data with standard error of the mean graphed as error bars. Supplementary Table S1 lists the primers used in this study along with their sequences. For the radiolabeled RT-PCR, the forward primer of lncRNA-CMPK2 was 5′ labeled with 32P using Optikinase (United States Biochemicals). Reverse transcription was performed as described above. The PCR reaction was performed using PrimeSTAR GXL DNA polymerase and the products were loaded on a 5% native polyacrylamide gel electrophoresis (PAGE) along with a radiolabeled size marker.

### Western blotting

The samples were boiled after the addition of equal volume of loading buffer (0.25 M Tris-HCl pH 6.8, 10% 2-mercaptoethanol, 4% sodium dodecyl sulfate (SDS), 10% sucrose, 0.004% Bromophenol blue) and were loaded onto a 10% SDS-PAGE. The proteins were transferred to polyvinylidene difluoride membranes (Thermo Scientific). The membranes were blocked with 2% bovine serum albumin (Santa Cruz) and incubated with primary antibodies. Subsequently, the membranes were incubated with secondary antibodies. Antibody incubations were performed for 1 h at room temperature in Can Get Signal (TOYOBO, Takara scientific) solution. The membranes were incubated with SuperSignal West Pico (Pierce) and visualized using LAS-2000 analyzer (GE).

### *In vitro* transcription, RNA transfection and HCV infection

HCV infection was performed as previously described with slight modification (35,38,59). Briefly, the plasmid pJFH1 was linearized with XbaI and treated with Mung bean exonuclease. The linearized DNA was transcribed *in vitro* by using a MEGAscript T7 kit (Applied Biosystems) according to the manufacturer's protocol. The *in vitro*-transcribed RNA was transfected into Huh7.5 cells. After several passages, supernatant was collected and infectious titer of the HCV viral particles was determined by the 50% tissue culture infectious dose (TCID_50_) assay. Cells were infected with HCV JFH1 viral particles at multiplicity of infection (MOI) of 0.1 for 2 h. Next, the media was changed to fresh media and IFN-α was added at 500 units/ml. After 24 h, the RNA was harvested and analyzed by RT-qPCR.

### Knockdown studies

The lentiviral expression vectors containing the shRNA constructs (Sigma) and lentiviral packaging plasmid mix were cotransfected into Lenti-X 293T cells (Clontech) and the supernatants were collected 48 h after transfection. The culture supernatants were centrifuged at 1000 × g for 10 min and cleared through 0.45 μm pore size filter. The infectious supernatant was used to infect cells, which were subjected to selection by puromycin to eliminate non-transfected cells prior to being used in experiments.

### Patient samples

Subjects undergoing liver transplantation for end-stage disease included those with chronic HCV infection (HCV antibody and serum RNA positive), autoimmune hepatitis, α-1 antitrypsin deficiency, alcohol-related liver disease, primary sclerosing cholangitis and non-alcoholic steatohepatitis. Subjects provided written informed consent for use of native liver explant, conforming to the ethical guidelines of the 1975 Declaration of Helsinki with prior approval of the institutional review board for human studies at Henry Ford Health System and University Hospitals of Cleveland. Liver explant tissue sections were snap frozen. Frozen liver samples were homogenized with tissue grinder in PBS on ice. The samples were centrifuged at 3000 rpm for 10 min to remove the cell debris. Subsequently the supernatant was subjected to RNA extraction as described above followed by RT-qPCR reaction. We confirmed the presence and absence of HCV genome in samples from HCV-infected and non-HCV-infected donors, respectively, using RT-qPCR (Supplementary Table S3). An independent set of liver samples were obtained from the Biobank of the University of Navarra under approval from the Ethical and Scientific Committees. Total RNA from frozen samples was extracted in 1 ml of TRIZOL using the homogenizer ULTRA-TURRAX (t25 basic IKA-WERKE).

## RESULTS

### Identification of IFN-stimulated lncRNAs

**Figure 1.**
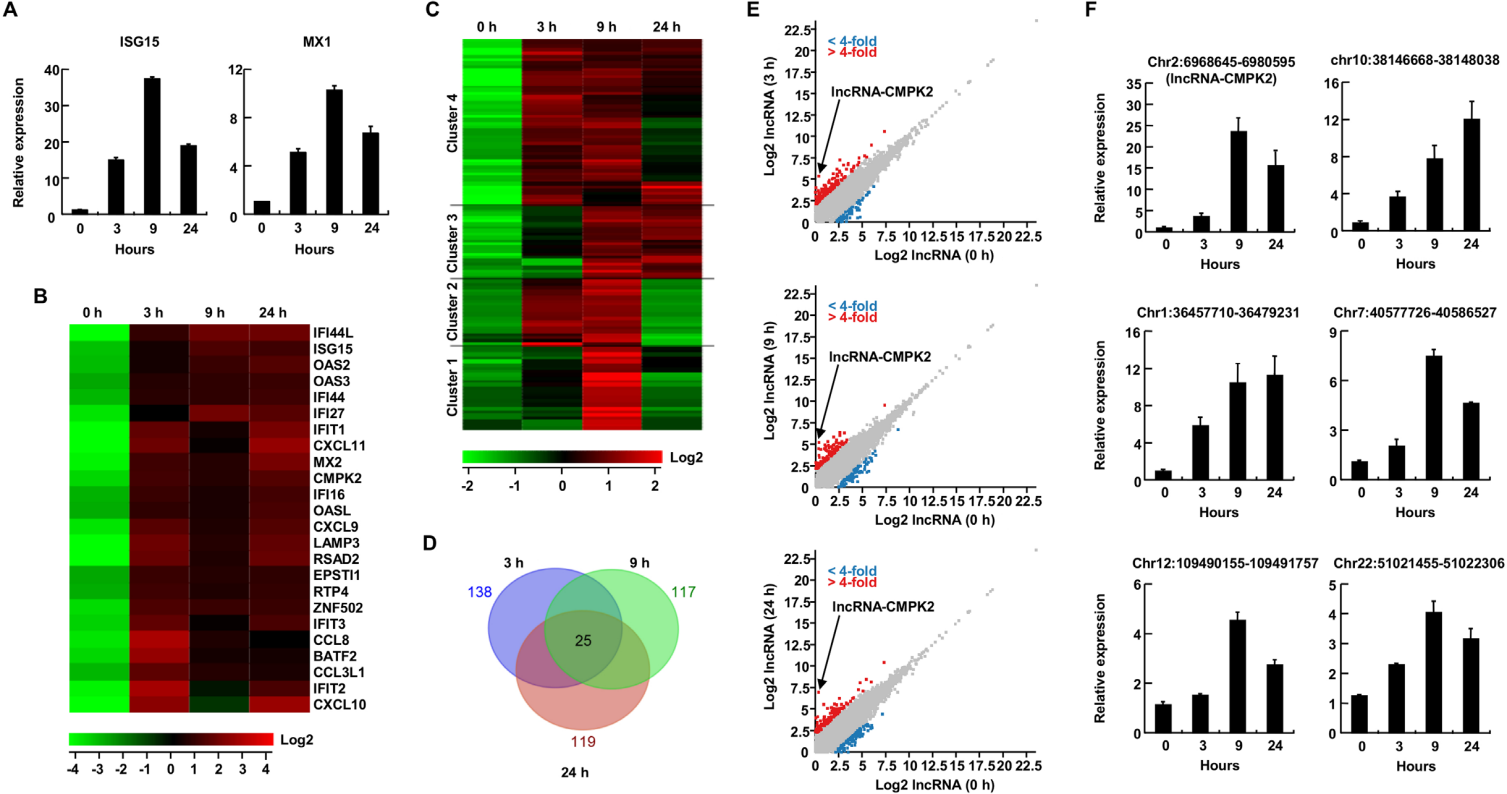
IFN-α induces hundreds of protein coding and long non-coding RNAs in human hepatocytes. (**A**) Induction of expression of ISG15 and Mx1 mRNAs at three time points following IFN-α addition in primary human hepatocytes measured by RT-qPCR. In this and the following panels, results are averages for at least two independent biological duplicate experiments with a minimum of two technical replicates per experiment. Error bars represent standard error of the mean. (**B**) Expression profiles of protein-coding RNAs that show over 10-fold change in expression following 3, 9 and 24 h of IFN-α treatment compared to untreated cells (samples shown as 0 h time point). The color scale is shown at the bottom. (**C**) Heat map and (**D**) Venn diagram of putative lncRNAs showing 4-fold or greater change in expression following IFN-α treatment. The time points shown are identical to those in panel (B). The color scale is shown at the bottom of heat map. (**E**) Scatter plots depicting the annotated putative lncRNAs that show a statistically significant change of 4-fold or more after 3 (top), 9 (middle) and 24 h (bottom) of IFN stimulation. The gray dots mark putative lncRNAs that did not show a significant change in expression. Red and blue dots correspond to upregulated and downregulated putative lncRNAs, respectively. The location of lncRNA-CMPK2 is shown. (**F**) RT-qPCR analysis of representative lncRNAs at indicated time points following IFN-α treatment. Strand-specific RT was performed using lncRNA-specific primers to ensure the specificity of detection, followed by qPCR. The locus of each analyzed lncRNA is shown on top. Numbers at the bottom refer to time point after IFN stimulation.

To gain insight into the potential role of the non-coding transcriptome in the IFN response, we performed high-throughput RNA sequencing on total cellular RNA obtained from IFN-α-stimulated human primary hepatocytes. As mentioned above, IFN-α induces a highly effective antiviral response against hepatitis resulting from HCV infection, and thus hepatocytes provide an ideal model system for functional analysis of IFN-mediated antiviral activity. Primary hepatocytes were obtained from five donors of both genders ranging from 10 months to 57 years of age (Supplementary Figure S1A). We selected donors who were negative for HIV, HBV, HCV and CMV infections and had no history of alcohol abuse or other conditions that may affect liver function (Supplementary Figure S1A) to ensure that the observed gene expression patterns were not affected by these conditions. The cells demonstrated high viability and were treated with 500 units/ml of IFN-α for 3, 9 and 24 h prior to harvest of total cellular RNA for sequencing. We also extracted RNA from mock-treated cells that had not received IFN-α as a control. Using RT-qPCR, we confirmed that the known protein-coding ISGs Mx1 and ISG15 were significantly upregulated in each of the five individual donor samples (Supplementary Figure S1B and C), and in pooled samples containing equal amounts of RNA from each donor (Figure 1A). These results indicated that the primary hepatocytes used in this study exhibited an appropriate biological response to IFN-α.

We performed paired-end strand-specific RNA-seq on the pooled control and IFN-treated samples used in Figure 1A. Analysis of the sequencing results indicated that several protein-coding genes were upregulated over 10-fold in IFN-treated cells compared to untreated controls (Figure 1B). Consistent with previous reports, known ISGs, such as viperin (RSAD2), IFIT1, IFIT2, IFIT3 and Mx2 showed a significantly increased expression in sequencing results from IFN-treated samples compared to controls. Analysis of the expression changes in transcripts annotated as putative lncRNAs in public databases indicated that 138, 117 and 119 lncRNAs showed a statistically significant upregulation of 4-fold or more in IFN-α-treated cells at 3, 9 and 24 h time points, respectively (Figure 1C and D, Supplementary Figure S2A–C and Table S2). Of these, 25 lncRNAs showed more than 4-fold upregulation across all time points (Figure 1D). While in this study we focused our analysis on the putative lncRNAs that were upregulated in response to IFN stimulation, we could detect several annotated putative lncRNAs that were downregulated over 4-fold as a result of IFN stimulation at one or more of the three analyzed time points (Figure 1E). Although the identified IFN-stimulated lncRNAs have been annotated as putative lncRNAs by large-scale transcriptome analyses in public databases, to our knowledge none of them has been functionally studied to date. Clustering analysis indicated that in terms of temporal pattern of expression, the upregulated lncRNAs fell into several categories that differ in the onset of upregulation (early versus late) and its duration (Figure 1C and Supplementary Figure S2A–C), potentially offering clues into their mode of induction and function. We confirmed the IFN-mediated upregulation of a subset of the annotated putative lncRNAs using RT-qPCR in the IFN-treated primary human hepatocytes (Figure 1F). Together, these results demonstrated that type I IFN induces over a hundred lncRNAs in addition to protein-coding genes in primary human hepatocytes.

### lncRNA-CMPK2 is highly upregulated in response to IFN-α treatment

**Figure 2.**
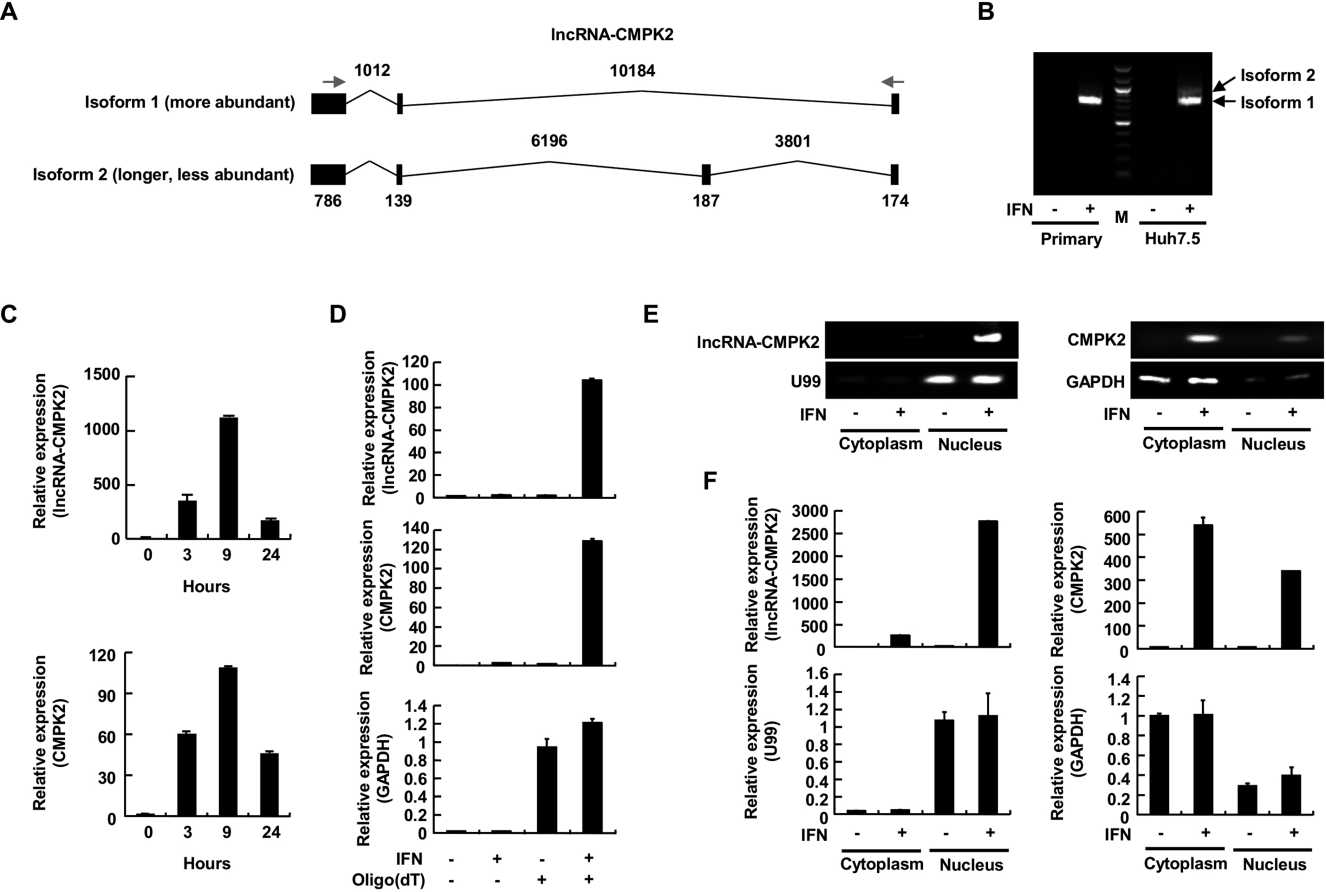
Characterization of lncRNA-CMPK2. (**A**) The genomic architecture of lncRNA-CMPK2. Exons and introns are shown as filled rectangles and thin lines, respectively. The two splicing isoforms of the RNA are shown. The numbers at the top and bottom indicate the size of introns and exons in nucleotides, respectively. (**B**) RT-PCR assays indicating the presence of the two isoforms of lncRNA-CMPK2 in Huh7.5 hepatocyte cell line, with the shorter isoform being more abundant. The longer isoform is not detectable in primary human hepatocytes (lanes marked Primary). Plus and minus signs indicate the presence and absence of IFN treatment. (**C**) The temporal induction pattern of lncRNA-CMPK2 and its neighboring CMPK2 protein-coding RNA in Huh7.5 cells at three time points after IFN-α treatment (shown below each graph) measured by RT-qPCR. In this and the following panels, results are averages for at least two independent biological duplicate experiments with a minimum of two technical replicates per experiment. Error bars represent standard error of the mean. (**D**) lncRNA-CMPK2 is polyadenylated. RNA extracted from Huh7.5 cells is used in RT reactions with oligo(dT) primers followed by qPCR using primers specific to lncRNA-CMPK2 (top), CMPK2 (middle) and GAPDH (bottom). + and – signs at the bottom indicate the included or omitted ingredient. (**E** and **F**) lncRNA-CMPK2 is a nuclear transcript. Lanes marked nucleus or cytoplasm contain the RNA extracted from nuclear and cytoplasmic fractions obtained from Huh7.5 cells, respectively. Expression of lncRNA-CMPK2, CMPK2, U99 (nuclear marker) and GAPDH (cytoplasmic RNA) in the nuclear and cytoplasmic fractions were detected by RT-PCR followed by agarose gel electrophoresis (E) or RT-qPCR (F). Identity of the genes detected in each gel or graph is shown to the left of the gel or in the *X* axis label. + and – signs at the bottom indicate the presence or absence of IFN treatment.

Among the identified lncRNAs, an RNA annotated as AC017076.5 which maps to chr2p25.2 (hg19, chr2:6,968,644-6,980,595, Supplementary Figure S3A) showed the highest level of induction after IFN stimulation and was thus chosen for further analysis. AC017076.5 is a multiexonic transcript positioned downstream of the known protein-coding ISG CMPK2 in a non-overlapping, head to tail orientation (Figure 2A and Supplementary Figure S3A). Considering this proximity, we named this transcript lncRNA-CMPK2. We confirmed the induction of lncRNA-CMPK2 in response to IFN-α in Huh7.5 hepatocytes, which showed a very strong induction based on RT-qPCR (Figure 2B and C). Analysis of the functional motifs near the locus of the RNA indicated the presence of an IFN-stimulated response element ∼4 kb upstream of the transcriptional start site of lncRNA-CMPK2. We could detect the expression of two alternatively spliced isoforms for lncRNA-CMPK2 in Huh7.5 cells with the shorter isoform being more abundant (Figure 2A and B). By performing qPCR assays on cDNAs made using oligo(dT) primers, we could show that lncRNA-CMPK2 is polyadenylated (Figure 2D). PCR reactions on cDNAs made in mock reverse transcription reactions that lacked oligo(dT) primers did not result in a detectable qPCR signal (Figure 2D). 3′ RACE analysis indicated the presence of a single polyadenylation site at the 3′ end, which was located ∼350 nucleotides downstream of the annotated 3′ end of the transcript (Supplementary Figure S3A and B). We analyzed the sequence of the two isoforms of lncRNA-CMPK2 to define the location of the exon–intron junctions in both isoforms and observed that the longer isoform results from the inclusion of a cassette exon that is absent in the shorter, more abundant isoform (Figure 2A). Analysis of the annotation databases indicated that the longer isoform is a novel, previously unreported transcript. In addition to the main isoform, another isoform resulting from alternative promoter and splice site usage has been annotated in public databases; however, we could not detect the presence of this latter transcript in our samples (data not shown). To define if either of the two isoforms detected in Figure 2B had any protein-coding potential, we analyzed them for the presence of ORFs. Neither transcript had an ORF longer than 69 amino acids (Supplementary Figure S3C–E and data not shown). The existing short ORFs lacked the Kozak sequence and were positioned such that translation would most likely trigger the nonsense-mediated decay pathways due to the presence of very long 3′UTRs and upstream ORFs (Supplementary Figure S3C–E). Further, analysis of the pattern of their phylogenetic conservation indicated the presence of frameshift-inducing insertion/deletions and sequence variations that were not consistent with conservation of protein-coding capacity and resulted in a high ratio of non-synonymous to synonymous codon changes (Supplementary Figure S3F). As the longer isoform detected in Figure 2B was present in very low abundance in the Huh7.5 cells and was not detectable in primary human hepatocytes, we focused the rest of our study on the shorter isoform which was also by far the more abundant one.

We analyzed the subcellular localization of the main isoform of lncRNA-CMPK2 using nuclear and cytoplasmic fractions obtained from Huh7.5 hepatocytes followed by RT-qPCR-based detection. As can be seen in Figure 2E and F and Supplementary Figure S3G, the vast majority of lncRNA-CMPK2 transcripts were nuclear, while the protein-coding ISGs CMPK2 and viperin mRNAs were mainly cytoplasmic. These results also indicated that lncRNA-CMPK2, which was transcribed from a locus close to the loci of protein-coding genes CMPK2 and viperin (Supplementary Figure S3A), was an independent transcript and did not co-localize with its neighboring mRNAs. Further, the nuclear localization of lncRNA-CMPK2 further confirmed that it was indeed a non-protein-coding transcript. Taken together, these results indicated that lncRNA-CMPK2 was an IFN-induced nuclear long non-protein-coding RNA.

### lncRNA-CMPK2 is a bona fide ISG induced by both type I and type II IFNs

**Figure 3.**
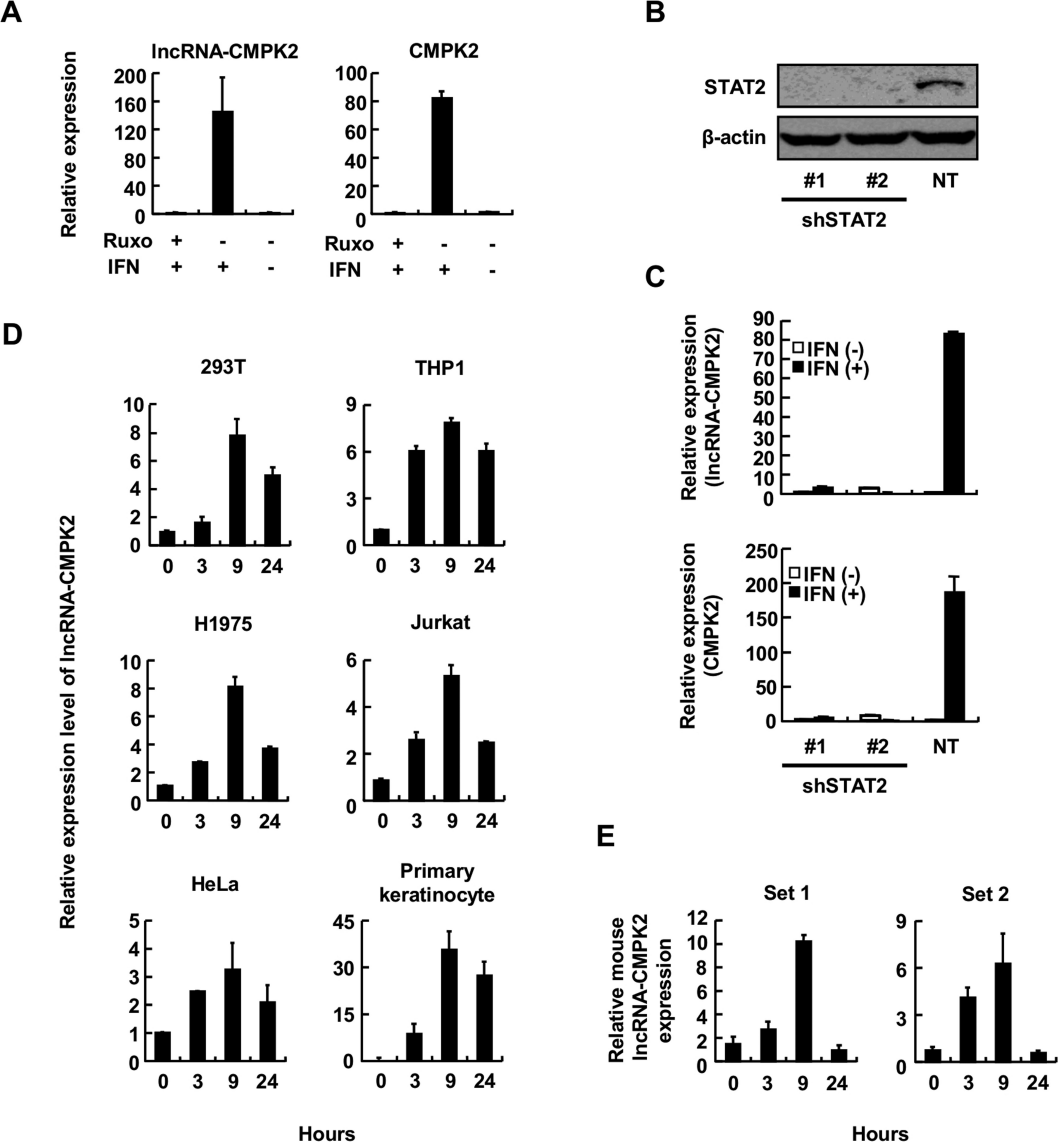
lncRNA-CMPK2 is a bona fide ISG and is induced by IFN in diverse cell types in human and mouse. (**A**) Induction of lncRNA-CMPK2 by IFN is mediated through the JAK pathway. RT-qPCR assays to detect lncRNA-CMPK2 (left) or CMPK2 (right) were performed on cellular RNA from Huh7.5 cells treated with JAK inhibitor Ruxolitinib (Ruxo). + and – signs at the bottom indicate the added or omitted ingredient, respectively. In this and the following panels, results are averages for at least two independent biological duplicate experiments with a minimum of two technical replicates per experiment. Error bars represent standard error of the mean. (**B**) Western blot assay on total cellular protein extracted from cells transfected with one of the two shRNA constructs against STAT2 (shSTAT2) or a non-targeting, control shRNA construct (NT). The specificity of the antibody used is shown to the left. (**C**) IFN-induced upregulation of lncRNA-CMPK2 is mediated through STAT2. RT-qPCR assays were performed on RNA from STAT2-knockdown cells (shSTAT2) and control cells transfected with a non-targeting shRNA (NT) to detect the level of lncRNA-CMPK2 (top) or CMPK2 (bottom). (**D**) lncRNA-CMPK2 is induced by IFN in diverse cell types. The identity of the cell type tested is indicated above each panel. Numbers at the bottom indicate the time point after addition of IFN-α (500 units/ml) in hours. (**E**) IFN-mediated induction of lncRNA-CMPK2 is conserved between mouse and human, as shown by RT-qPCR assays on IFN-treated mouse myoblast C2C12 cells. Numbers at the bottom of graphs indicate the time point after IFN addition in hours. To ensure the specificity of the signal, two different primer sets against the putative mouse lncRNA-CMPK2 ortholog were used.

The IFN-stimulated induction of previously-investigated, protein-coding ISGs is mediated through the JAK-STAT signaling pathway (32,33). Binding of IFN-α to its receptor results in phosphorylation of JAK proteins and the receptor itself, which in turn leads to recruitment and phosphorylation of the transcription factors STAT1 and STAT2 and their translocation into the nucleus (32,33). To determine whether the expression of lncRNA-CMPK2 was similarly dependent on the JAK-STAT pathway, we treated the Huh7.5 hepatocytes with IFN-α along with the commonly used JAK inhibitor, ruxolitinib. RT-qPCR analyses indicated that ruxolitinib almost completely abrogated the transcriptional upregulation of lncRNA-CMPK2 along with known protein-coding ISGs such as its neighboring ISG, CMPK2 (Figure 3A). To further confirm this result, we used a shRNA-mediated knockdown strategy to reduce the cellular level of STAT2 (Figure 3B). During the IFN response, together with STAT1 and IRF9, STAT2 binds the promoter of ISGs to induce transcription. Consistent with the results from the use of the JAK inhibitor, lncRNA-CMPK2 upregulation by IFN-α was almost completely blocked in STAT2-knockdown Huh7.5 cells (Figure 3C). It is known that many ISGs are induced by both type I and type II IFNs (40,43). To determine if lncRNA-CMPK2 is similarly responsive to type II IFN, Huh7.5 cells were treated with IFN-γ for 3, 9 and 24 h followed by analysis of lncRNA-CMPK2 expression using RT-qPCR. Similar to many studied ISGs, lncRNA-CMPK2 was strongly induced by IFN-γ (Supplementary Figure S4A). However, treating cells with TNF-α, a non-IFN cytokine that acts through activation of the NF-kB pathway, did not result in induction of lncRNA-CMPK2 (Supplementary Figure S4B). In contrast, the level of CXCL10, which is known to be induced by both IFN and TNF-α showed the expected upregulation under these conditions (Supplementary Figure S4B). Together, these results indicated that lncRNA-CMPK2 was a bona fide ISG and was specifically induced by the JAK-STAT pathway.

We could also show that the induction of lncRNA-CMPK2 in response to IFN treatment is not restricted to cells of liver origin, as in addition to Huh7.5 cells and primary hepatocytes, it is induced after IFN-α treatment in several human cell lines such as HEK293T (kidney origin), THP1 (monocyte origin), H1975 (lung epithelial origin), Jurkat (T cell origin), HeLa (cervical origin) and primary human keratinocyte (Figure 3D). Analysis of the phylogenetic conservation of the RNA showed that lncRNA-CMPK2 is highly conserved among primates. Similarly, about half of the exonic sequences in lncRNA-CMPK2 are conserved among mammals with syntenic conservation of the locus between mouse and human; however, it is not conserved in non-mammalian organisms (Supplementary Figure S4C and D). To determine if the induction of lncRNA-CMPK2 in response to IFN was conserved, we treated mouse C2C12 myoblast cells with IFN-α followed by RT-qPCR using two different sets of primers that targeted the region conserved between mouse and human. Interestingly, we were able to detect the upregulation of putative mouse lncRNA-CMPK2 in response to IFN stimulation (Figure 3E). These results indicated that IFN-mediated induction of lncRNA-CMPK2 is likely conserved among mammals and is not tissue-specific, but rather part of the global transcriptional response to IFN stimulation.

### lncRNA-CMPK2 is a negative regulator of the IFN response

**Figure 4.**
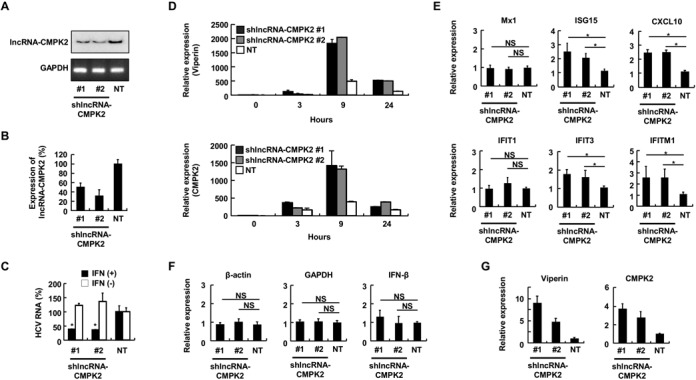
lncRNA-CMPK2 is a negative regulator of the IFN pathway. (**A**) Basal level of lncRNA-CMPK2 in Huh7.5 cells stably transfected by one of the two targeting shRNA vectors (lanes marked #1 and #2) or a control, non-targeting shRNA (NT). Expression of lncRNA-CMPK2 in the absence of IFN was detected by RT-PCR using radiolabeled primers followed by analysis on PAGE (top panel). Expression of GAPDH was detected by RT-PCR followed by agarose gel electrophoresis (bottom panel). (**B**) RT-qPCR assays on cells analyzed in panel (A) after 24 h of IFN-α treatment. In this and the following panels, results are averages for at least two independent biological duplicate experiments with a minimum of two technical replicates per experiment. Error bars represent standard error of the mean. (**C**) Knockdown of lncRNA-CMPK2 results in a significant decrease in replication of HCV in the presence of IFN. RT-qPCR assays were performed on RNA from lncRNA-CMPK2 stable knockdown Huh7.5 cells that had been infected with HCV JFH1 at an MOI of 0.1 for 2 h followed by IFN-α treatment for 24 h (black columns) or mock treatment (white columns). In this and the following panels, asterisks indicate a significant difference from the control as calculated by Student's *t*-test (*P* < 0.05). NS: not significant (**D**) Time course of induction of ISGs by IFN in control (NT) and lncRNA-CMPK2-knockdown cells. The level of viperin (top) and CMPK2 (bottom) is determined before (0 h), and 3, 9 and 24 h after IFN treatment. Numbers at the bottom indicate the time point in hours. (**E**) lncRNA-CMPK2 knockdown results in a rise in expression of several ISGs. The level of ISGs after 9 h of IFN stimulation of Huh7.5 cells is shown. (**F**) lncRNA-CMPK2 knockdown does not affect housekeeping genes or the endogenous IFN-β gene expression. The level of expression after 9 h of IFN stimulation of Huh7.5 cells is shown. (**G**) The level of viperin and CMPK2 are raised in lncRNA-CMPK2-knockdown cells even in the absence of IFN stimulation.

The conservation pattern of lncRNA-CMPK2 and its induction in response to IFN-α in diverse cell types suggested that it may play a functional role in the IFN response. To investigate this possibility, we used a shRNA-mediated knockdown approach. Of the five tested shRNA constructs, only two could effectively reduce the level of the lncRNA both before and after its strong induction by IFN-α stimulation (Figure 4A and B). As control, cells were transduced in parallel with a non-targeting shRNA construct. To determine if the reduced level of lncRNA-CMPK2 had a functional impact on the IFN response, we infected control and knockdown cells with JFH1 HCV, which is known to be sensitive to IFN-α treatment, followed by measuring the level of HCV genome after IFN stimulation. Intriguingly, we observed a significant reduction in the level of HCV genomic RNA in the knockdown cells compared to control (Figure 4C and Supplementary Figure S5). In the absence of IFN stimulation, however, knockdown and control cells had similar levels of HCV genomic RNA (Figure 4C and Supplementary Figure S5), raising the possibility that the observed effect involved attenuation of the antiviral activity of IFN response on HCV replication.

To determine if the impact of lncRNA-CMPK2 knockdown on HCV replication was mediated through direct modulation of the IFN response, we analyzed the IFN-stimulated expression level of a number of protein-coding ISGs with known antiviral activity against HCV (60–62). Surprisingly, the level of the majority of tested ISGs showed an increase in lncRNA-CMPK2-knockdown cells (Figure 4D and E). In contrast, the level of a subset of tested ISGs including Mx1 and IFIT1, and housekeeping genes such as β-actin and GAPDH or the endogenous IFN-β remained unchanged (Figure 4E and F). Among the analyzsed ISGs, the genomic loci of CMPK2 and viperin neighbor the locus of lncRNA-CMPK2 (Supplementary Figure S3A), while IFIT3, IFIT1, IFIT1M, Mx1, CXCL10 and ISG15 are located on different chromosomes and thus, the impact of knockdown of lncRNA-CMPK2 must be mediated through a trans-acting mechanism. As lncRNA-CMPK2 is a nuclear transcript, it is likely that it exerts its action through regulation of a nuclear event. Comparison of the IFN induction of viperin and CMPK2 in knockdown and control cells indicated that, although the most prominent difference was observed after 9 h of IFN-α stimulation, the magnitude of their induction was also increased at earlier and later time points (Figure 4D). Surprisingly, even in the absence of IFN-α, the basal levels of viperin and CMPK2 were higher in lncRNA-CMPK2-knockdown cells (Figure 4G), suggesting that the reduction in the basal level of lncRNA-CMPK2 (Figure 4A) led to induction of expression of these two ISGs under both basal and IFN-stimulated conditions.

**Figure 5.**
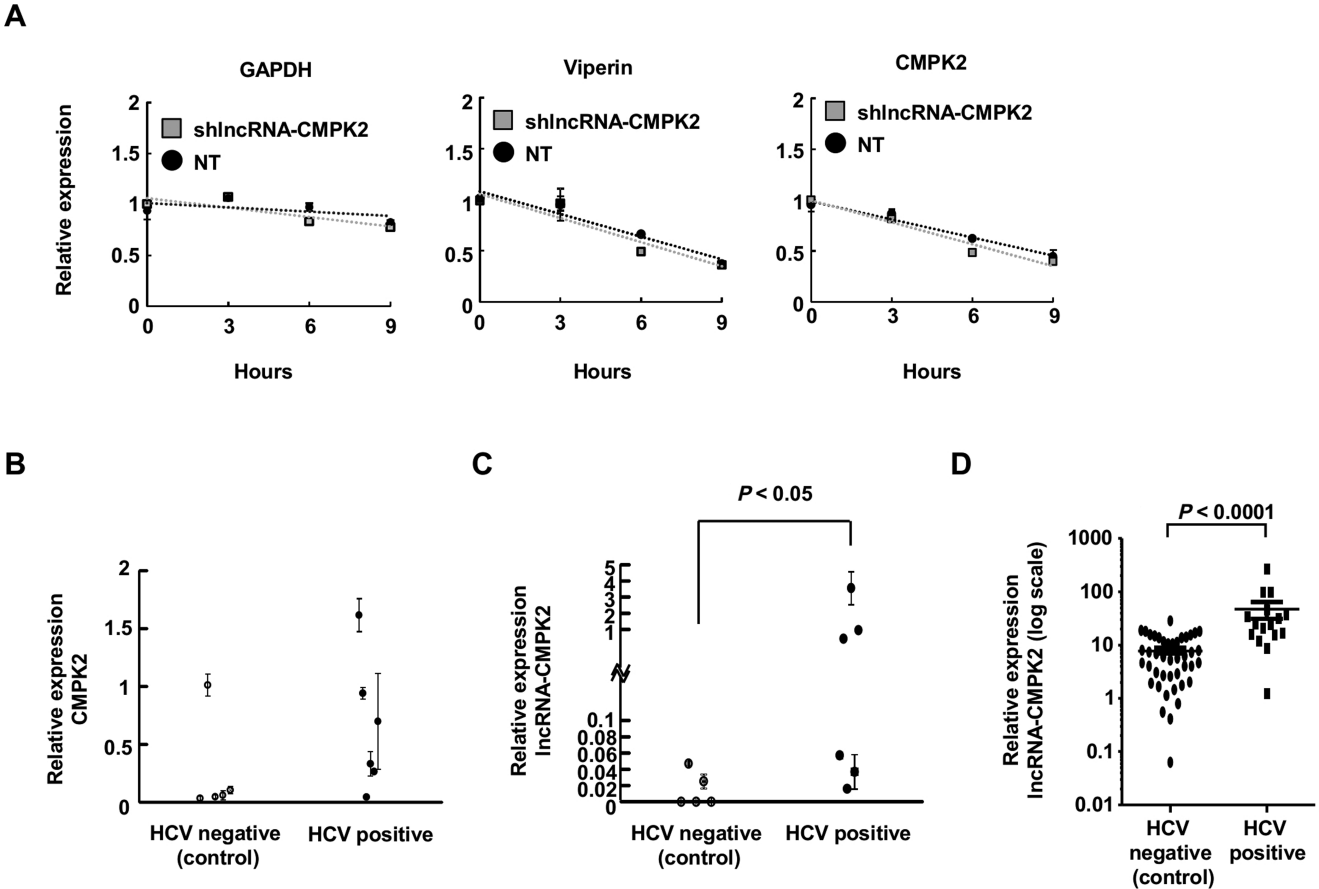
Transcriptional regulation of the IFN response by lncRNA-CMPK2. (**A**) lncRNA-CMPK2 knockdown does not affect the stability of the ISGs. The steady-state level of GAPDH, viperin and CMPK2 mRNAs are determined by RT-qPCR at the indicated time points following blockage of Pol II transcription by actinomycin D in IFN-stimulated control transfected with non-targeting shRNAs (NT) or lncRNA-CMPK2-knockdown cells. (**B**, **C**) The levels of CMPK2 (B) and lncRNA-CMPK2 (C) were measured in liver samples from HCV-infected donors compared to non-HCV-infected controls by RT-qPCR. In HCV-positive group *n* = 6; in HCV-negative group *n* = 5. (**D**) Analysis of the level of lncRNA-CMPK2 in an independently-obtained and processed second set of HCV-infected and non-infected human liver samples confirmed the induction of lncRNA-CMPK2 in response to HCV infection *in vivo*. Horizontal bars indicate the average value of the datapoints in each group with the standard error of the mean as error bars. In HCV-positive group *n* = 16; in HCV-negative group *n* = 43. The *Y* axis is in log scale. The HCV profile of each group of donors is shown below the graph. Statistical significance in (C) and (D) panels was determined by Mann–Whitney *U* test.

To determine whether the rise in the level of ISGs caused by lncRNA-CMPK2 knockdown was the result of transcriptional induction or increased stability, we blocked RNA Pol II transcription using actinomycin D, followed by monitoring the level of the two RNAs and GAPDH as control at several time points. We could show that both viperin and CMPK2 mRNAs were stable RNAs with estimated half-lives of 6–9 h (Figure 5A). Importantly, the half-lives of viperin and CMPK2 were not significantly changed in lncRNA-CMPK2-knockdown cells compared to control, indicating that the observed rise in the level of these ISGs was transcriptional (Figure 5A). Together, these results suggest that reducing the cellular level of lncRNA CMPK2 by shRNA-mediated knockdown results in transcriptional upregulation of a subset of ISGs both under basal conditions and after IFN stimulation. Thus, lncRNA-CMPK2 has an inhibitory effect on transcription of IFN-stimulated antiviral genes.

### lncRNA-CMPK2 is upregulated in liver samples from HCV-infected patients

Finally, to determine if lncRNA-CMPK2 may potentially play a similar regulatory role *in vivo*, we tested the expression of the RNA in liver samples from non-HCV-infected donors and donors with chronic HCV infection, which is known to lead to activation of the IFN response (60). As expected, we could confirm the presence of the HCV genome and activation of the IFN response in the HCV-infected donors (Figure 5B, and Supplementary Table S3). Interestingly, the level of lncRNA-CMPK2 was significantly higher in liver tissue from a subset of HCV-infected patients compared to controls (Figure 5C), suggesting that lncRNA-CMPK2 may play a similar regulatory role in response to viral infections *in vivo*. To ensure reproducibility, we repeated the above analysis on a second set of liver samples that were processed and analyzed in an independent manner. Importantly, these experiments also showed an increase in the level of lncRNA-CMPK2 in HCV-infected liver samples compared to non-HCV samples (Figure 5D), confirming the induction of lncRNA-CMPK2 in response to HCV infection *in vivo*.

## DISCUSSION

We have shown that the cellular level of a large number of human long non-coding transcripts are altered during the IFN response. Functional analysis of one of the most highly induced lncRNAs, which we named lncRNA-CMPK2, indicated that it acts as a negative transcriptional regulator of a subset of IFN-induced genes. Prevention of excessive or uncontrolled IFN response is critical for preventing inflammatory damage, and several proteins are known to negatively regulate the IFN response at the level of IFN receptor, production of endogenous IFN or activation of the JAK-STAT pathway (63–66). A recent report by Li *et al*. has shown that a protein-coding ISG, ASCC3, has a mild negative regulatory effect on the expression of a number of ISGs by regulating transcription factors IRF3 and IRF7 (44). Although the subset of ISGs that were regulated by ASCC3 do not correlate with those regulated by lncRNA-CMPK2, it is possible to envision that the RNA may similarly function by regulation of transcriptional factors involved in the IFN response. An alternative possibility is that the lncRNA may bind ASCC3 or similar negative regulatory factors and potentiate their function. Indeed, existing data indicate that many lncRNAs function through negative regulation of the expression of other genes via modulation of transcription or epigenetic mechanisms (3,26,27,67–69).

Our results indicate that knockdown of the lncRNA resulted in transcriptional upregulation of several ISGs both under basal conditions and after IFN stimulation, resulting in a significant decrease in HCV replication in knockdown cells. The impact of knockdown of lncRNA-CMPK2 on ISG levels under basal conditions indicates that its transcriptional regulatory function is independent of the IFN response, which is not consistent with potentiation of the function of another IFN-stimulated negative regulatory factor such as ASCC3. Rather, it is likely that similar to many other lncRNAs, lncRNA-CMPK2 forms RNA–protein interactions with transcription factors or chromatin remodeling complexes that are present in significant levels under basal cellular conditions in the absence of IFN. The lncRNA–protein interactions in turn result in a transcriptional or epigenetic repressive state at genomic targets, which include a subset of ISG loci (3,5,6,25–27).

Interestingly, it has been recently shown that the IFN response is epigenetically regulated through di-methylation of histone 3 lysine 9 by G9a (70). As lncRNA-CMPK2 is a nuclear RNA, it may play a role in modulation of epigenetic marks by G9a or other chromatin-modifying factors such as PRC-2 (71) at the loci of a subset of ISGs. Alternatively, it may inhibit transcription at its target loci through non-epigenetic mechanisms, such as interference with the formation of transcriptional complexes at the promoter of the target genes, or blocking the interaction of these promoters with enhancer regions that form the binding site for the STAT1/STAT2 dimers. Based on our results, we propose a model in which lncRNA-CMPK2 interacts with and helps guide chromatin modifying factors or transcriptional inhibitory factors to the locus of a subset of ISGs, resulting in a block to transcription or induction of a repressive chromatin state at these loci and negative regulation of the IFN response. Reducing the cellular level of the lncRNA results in loss of inhibition at these loci and transcriptional upregulation of both basal and induced expression. While the reduction in basal level of the lncRNA is sufficient to increase the basal transcription level at the target ISG loci, an impact on viral replication will not be observed until the level of ISGs is raised to functionally active levels by IFN stimulation. In the absence of shRNA-mediated knockdown, lncRNA-CMPK2 maintains a transcriptionally repressed state at its target loci, thus reducing the magnitude of the IFN response and likely helping to end the transcriptional induction cascade along with other negative inhibitory factors (44,63–66).

Taken together, our results indicate, for the first time, that some members of the human non-coding transcriptome are induced in response to IFN stimulation and, at least in the case of one IFN-induced lncRNA, this can lead to modulation of the expression of the known antiviral ISGs and a change in viral replication. While the detailed mechanism of function of this lncRNA as a negative regulator of the IFN response remains to be determined, the data presented above provide a first glimpse of the involvement of the long non-coding transcriptome in regulation of the IFN response.

## ACCESSION NUMBERS

RNA-seq data is deposited in GEO (SRP045406).

## SUPPLEMENTARY DATA

Supplementary Data are available at NAR Online.

SUPPLEMENTARY DATA
