# Supplementary material for: Negative regulation of the interferon response by an interferon-induced long non-coding RNA
Source: Nucleic Acids Res. 2014 Aug 13;42(16):10668–80. doi: 10.1093/nar/gku713 (PMC4176326; doi:10.1093/nar/gku713)
Supplement: SUPPLEMENTARY DATA [file supp_42_16_10668__index.html]

Negative regulation of the interferon response by an interferon-induced long non-coding RNA — SUPPLEMENTARY DATA 

# Negative regulation of the interferon response by an interferon-induced long non-coding RNA

## SUPPLEMENTARY DATA

**Files in this Data Supplement:**

- SUPPLEMENTARY DATA
- SUPPLEMENTARY DATA
- SUPPLEMENTARY DATA
- SUPPLEMENTARY DATA
