# Supplementary material for: Negative regulation of the interferon response by an interferon-induced long non-coding RNA
Source: Nucleic Acids Res. 2014 Aug 13;42(16):10668–80. doi: 10.1093/nar/gku713 (PMC4176326; doi:10.1093/nar/gku713)
Supplement: SUPPLEMENTARY DATA [file supp_gku713_nar-01057-r-2014-File007.pdf]

Supplementary information

for

Negative regulation of the interferon response by an interferon-induced long non-coding RNA

Hiroto Kambara, Farshad Niazi, Lenche Kostadinova, Dilip K. Moonka, Christopher T. Siegel, Anthony B. Post, Elena Carnero, Marina Barriocanal, Puri Fortes, Donald D. Anthony and Saba Valadkhan

Figure S1

A

No	Sex	Age (year)	Viability (%)	HIV	HCV	HBV	CMV	Alcohol	tabacco	Substance
1	Male	57	91.3	-	-	-	-	No	No	No
2	Female	52	96.7	-	-	-	-	No	No	No
3	Male	31	98.7	-	-	-	-	No	Yes	Yes
4	Female	10 months	81.2	-	-	-	-	No	No	No
5	Female	3	97.1	-	-	-	-	No	No	No

B

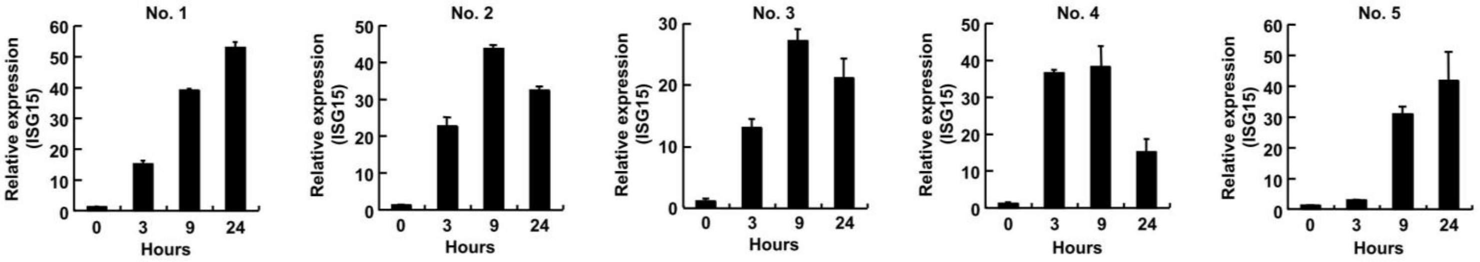

C

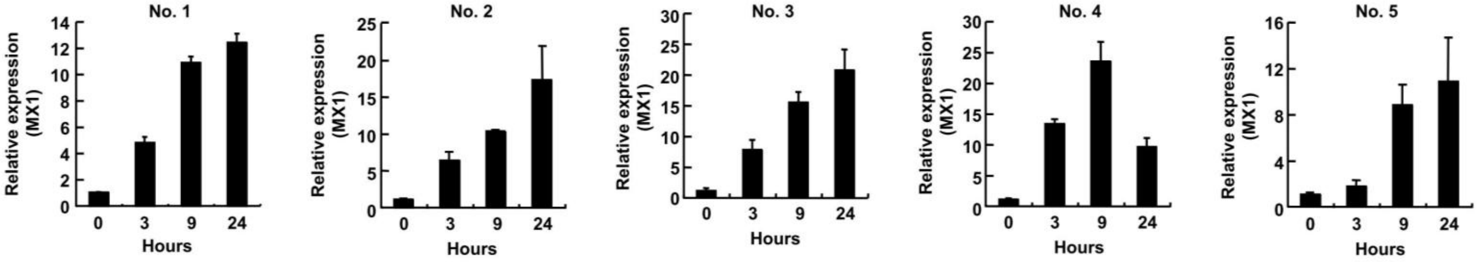

Figure S1. Primary human hepatocytes used in this study come from a diverse group of donors and show an appropriate response to IFN stimulation. (A) Characteristics of the donors. Minus signs indicate that the donors were not infected with the listed viruses. (B) and (C) RT-qPCR analysis of the expression of ISG15 (B) and Mx1 (C) mRNAs in primary hepatocytes from individual donors following IFN-α treatment (500 units/ml) for 3, 9 and 24 hours. Error bars represent standard error of the mean. The numbers at the bottom of each graph indicate the time point at which the cells were harvested following IFN treatment. Numbers on top refer to patient numbers shown in panel A.

Figure S2

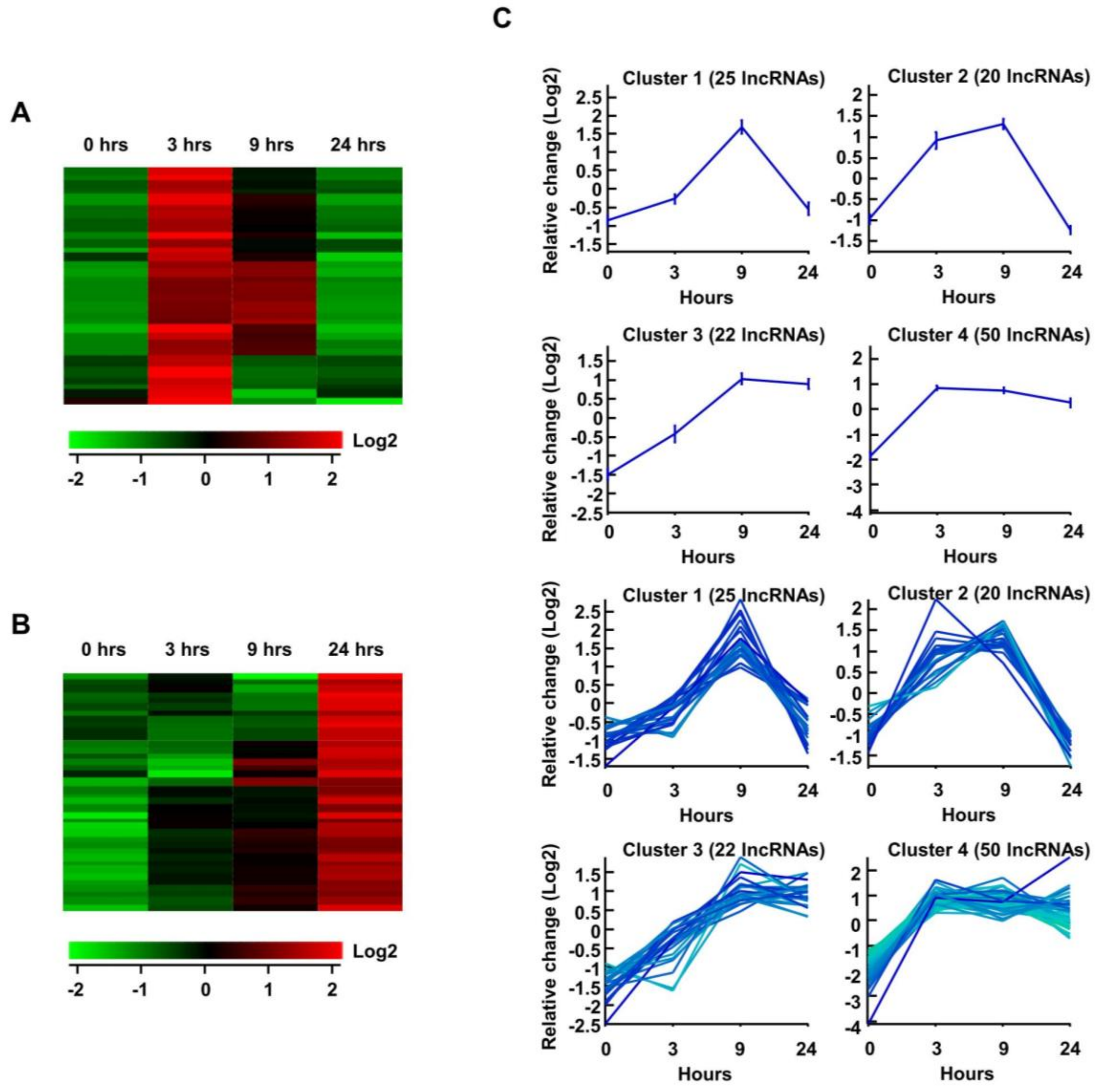

Figure S2. Expression pattern of IFN-induced lncRNAs. (A) and (B) Hierarchical clustering of annotated putative lncRNAs that are predominantly upregulated at 3 hours (A) or 24 hours (B) after IFN stimulation. For RNAs that are upregulated predominantly at 9 hour time point, see Fig. 1C. The time point after IFN stimulation is shown on top, with 0 hrs time point referring to untreated samples. Hrs: hours. (C). Temporal expression patterns of the lncRNAs that are predominantly upregulated after 9 hours of IFN stimulation (shown in Fig. 1C). The average of each group (Top 4 panels) and the expression pattern of individual RNAs (bottom 4 panels) are shown. The included RNAs show four fold or more induction at 9 hour time point and include the subset that are induced four fold or more at all three analyzed time points (see Fig. 1D).

Figure S3

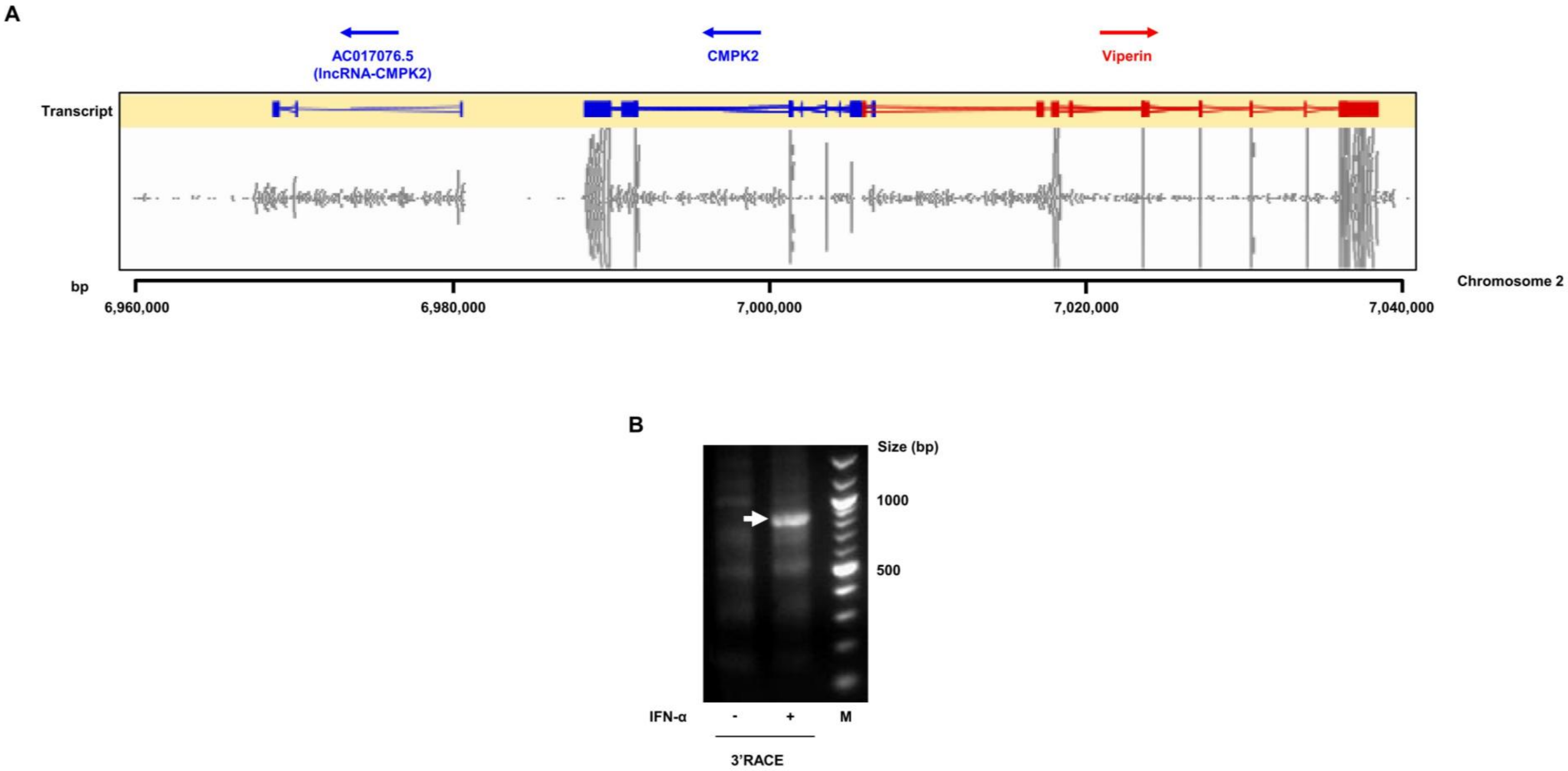

Figure S3. Characterization of lncRNA-CMPK2. (A) RNA-seq profile of lncRNA-CMPK2 locus and its neighboring ISG genes. The annotated exon/intron structure of genes is shown on top. The dots correspond to one 100 bp long RNA-seq read. Arrows indicate the orientation of transcription at each locus. (B) 3'-RACE（Rapid Amplification of cDNA Ends) to determine the 3' end of lncRNA-CMPK2. To distinguish the band corresponding to the 3' end of lncRNA-CMPK2 from non-specific bands, the experiment was performed in the presence and absence of IFN stimulation. Arrow points to the only band that shows differential amplification between the IFN treated and untreated samples. Sequencing of the DNA content of the band indicated that it indeed corresponded to the 3' end of the lncRNA-CMPK2.

Figure S3 (continued)

C

+1 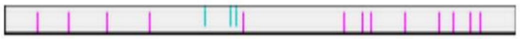

```
   1 ttcaatcccacccttccttaccagatactaaagtcatttctacctttcctcttgagattt
     F  N  P  T  L  P  Y  Q  I  L  K  S  F  L  P  F  L  L  R  F
  61 gctgttagatattagaacttctctttgtcacatacttacagggagattttgattatccat
     A  V  R  Y  *  N  F  S  L  S  H  T  Y  R  E  I  L  I  I  H
 121 gggattccattcataacctcataaaccacccccacgaagaaattatatatctcggtgaca
     G  I  P  F  I  T  S  *  T  T  P  T  K  K  L  Y  I  S  V  T
 181 tggttttctggtgccttggcaactgctcacgatgcatgggaagactaaacactttgtgta
     W  F  S  G  A  L  A  T  A  H  D  A  W  E  D  *  T  L  C  V
 241 cagcattctgtctcatccagtgaagacttcctctggattattgcagcagacacctctaac
     Q  H  S  V  S  S  S  E  D  F  L  W  I  I  A  A  D  T  S  N
 301 ctccttaatctgtactgaatcttgctctgtcgccaggctggagtgcagtggtgcgatatt
     L  L  N  L  Y  *  I  L  L  C  R  Q  A  G  V  Q  W  C  D  I
 361 ggctcactgcaacctccgcctctcgggttcaagcgattatcctgcctcagccttttgagt
     G  S  L  Q  P  P  P  L  G  F  K  R  L  S  C  L  S  L  L  S
 421 agctgggactacaggcacatggtaccatgccagactaattttttgtatttttagtagagat
     S  W  D  Y  R  H  M  V  P  C  Q  T  N  F  C  I  F  S  R  D
 481 gggattatcactatgttggccaagatggttttgatctcttgacttcgtgatccgccctcc
     G  I  I  T  M  L  A  K  M  V  L  I  S  *  L  R  D  P  P  S
 541 tcggcctcccaaagtgctgggatttcaggcgttagccaccgcacccagcctggacttgtc
     S  A  S  Q  S  A  G  I  S  G  V  S  H  R  T  Q  P  G  L  V
 601 tatttcatttggagaacttcaattgatctaaatctactttgcagcttttcattgttaatt
     Y  F  I  W  R  T  S  I  D  L  N  L  L  C  S  F  S  L  L  I
 661 ttgaatttatttaaacaagtaaaatatttaagacatttgtgtggaaaacctgtaacaaat
     L  N  L  F  K  Q  V  K  Y  L  R  H  L  C  G  K  P  V  T  N
 721 tgcatttggttcaatccctgattatgttttagatatgccaactctttttctctcttttttt
     C  I  W  F  N  P  *  L  C  F  R  Y  A  N  S  F  S  L  F  F
 781 taaaaatttgataaatactgaaaccaattcaatttagatcaaatacattacatttccact
     *  K  F  D  K  Y  *  N  Q  F  N  L  D  Q  I  H  Y  I  S  T
 841 agagggaggaaaagcgctaattttgaacctttatgagggaactgtgcttttgccgagata
     R  G  R  K  S  A  N  F  E  P  L  *  G  N  C  A  F  A  E  I
 901 atcacttccctcattacacacaaactgtgtctggaaaaacaacagtagttcctttcattt
     I  T  S  L  I  T  H  K  L  C  L  E  K  Q  Q  *  F  L  S  F
 961 ttatttaaatctcttctctaatgggaaaacaaaggacaaaacagggaacacttctaggtc
     L  F  K  S  L  L  *  W  E  N  K  G  Q  N  R  E  H  F  *  V
1021 aaagtagcaaatctttaaaagtggctaatttcttgcttcaagaattctaaatacagcccc
     K  V  A  N  L  *  K  W  L  I  S  C  F  K  N  S  K  Y  S  P
1081 cagggtcagaaaaatattc 1099
     Q  G  Q  K  N  I
```

D

******

+2 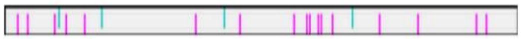

```
   2 tcaatcccacccttccttaccagatactaaagtcatttctacctttcctcttgagatttg
     S  I  P  P  F  L  T  R  Y  *  S  H  F  Y  L  S  S  *  D  L
  62 ctgttagatattagaacttctctttgtcacatacttacagggagattttgattatccatg
     L  L  D  I  R  T  S  L  C  H  I  L  T  G  R  F  *  L  S  M
 122 ggattccattcataacctcataaaccacccccacgaagaaattatatatctcggtgacat
     G  F  H  S  *  P  H  K  P  P  P  R  R  N  Y  I  S  R  *  H
 182 ggttttctggtgccttggcaactgctcacgatgcatgggaagactaaacactttgtgtac
     G  F  L  V  P  W  Q  L  L  T  M  H  G  K  T  K  H  F  V  Y
 242 agcattctgtctcatccagtgaagacttcctctggattattgcagcagacacctctaacc
     S  I  L  S  H  P  V  K  T  S  S  G  L  L  Q  Q  T  P  L  T
 302 tccttaatctgtactgaatcttgctctgtcgccaggctggagtgcagtggtgcgatattg
     S  L  I  C  T  E  S  C  S  V  A  R  L  E  C  S  G  A  I  L
 362 gctcactgcaacctccgcctctcgggttcaagcgattatcctgcctcagcctttttgagta
     A  H  C  N  L  R  L  S  G  S  S  D  Y  P  A  S  A  F  *  V
 422 gctgggactacaggcacatggtaccatgccagactaattttttgtatttttagtagagatg
     A  G  T  T  G  T  W  Y  H  A  R  L  I  F  V  F  L  V  E  M
 482 ggattatcactatgttggccaagatggttttgatctcttgacttcgtgatccgccctcct
     G  L  S  L  C  W  P  R  W  F  *  S  L  D  F  V  I  R  P  P
 542 cggcctcccaaagtgctgggatttcaggcgttagccaccgcacccagcctggacttgtct
     R  P  P  K  V  L  G  F  Q  A  L  A  T  A  P  S  L  D  L  S
 602 atttcatttggagaacttcaattgatctaaatctactttgcagcttttcattgttaattt
     I  S  F  G  E  L  Q  L  I  *  I  Y  F  A  A  F  H  C  *  F
 662 tgaatttatttaaacaagtaaaatatttaagacatttgtgtggaaaacctgtaacaaatt
     *  I  Y  L  N  K  *  N  I  *  D  I  C  V  E  N  L  *  Q  I
 722 gcatttggttcaatccctgattatgttttagatatgccaactctttttctctctttttttt
     A  F  G  S  I  P  D  Y  V  L  D  M  P  T  L  F  L  S  F  F
 782 aaaaatttgataaatactgaaaccaattcaatttagatcaaatacattacatttccacta
     K  N  L  I  N  T  E  T  N  S  I  *  I  K  Y  I  T  F  P  L
 842 gagggaggaaaagcgctaattttgaacctttatgagggaactgtgcttttgccgagataa
     E  G  G  K  A  L  I  L  N  L  Y  E  G  T  V  L  L  P  R  *
 902 tcacttccctcattacacacaaactgtgtctggaaaaacaacagtagttcctttcatttt
     S  L  P  S  L  H  T  N  C  V  W  K  N  N  S  S  S  F  H  F
 962 tatttaaatctcttctctaatgggaaaacaaaggacaaaacagggaacacttctaggtca
     Y  L  N  L  F  S  N  G  K  T  K  D  K  T  G  N  T  S  R  S
1022 aagtagcaaatctttaaaagtggctaatttcttgcttcaagaattctaaatacagccccc
     K  *  Q  I  F  K  S  G  *  F  L  A  S  R  I  L  N  T  A  P
1082 agggtcagaaaaatattc 1099
     R  V  R  K  I  F
```

Please see the next page for legend.

Figure S3 (continued)

E

+3 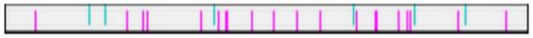

```
   3 caatcccacccttccttaccagatactaaagtcatttctacctttcctcttgagatttgc
     Q  S  H  P  S  L  P  D  T  K  V  I  S  T  F  P  L  E  I  C
  63 tgttagatattagaacttctctttgtcacatacttacagggagattttgattatccatgg
     C  *  I  L  E  L  L  F  V  T  Y  L  Q  G  D  F  D  Y  P  W
 123 gattccattcataacctcataaaccaccccacgaagaaattatatatctcggtgacatg
     D  S  I  H  N  L  I  N  H  P  H  E  E  I  I  Y  L  G  D  M
 183 gttttctggtgccttggcaactgctcacgatgcatgggaagactaaacactttgtgtaca
     V  F  W  C  L  G  N  C  S  R  C  M  G  R  L  N  T  L  C  T
 243 gcattctgtctcatccagtgaagacttcctctggattattgcagcagacacctctaacct
     A  F  C  L  I  Q  *  R  L  P  L  D  Y  C  S  R  H  L  *  P
 303 ccttaatctgtactgaatcttgctctgtcgccaggctggagtgcagtggtgcgatattgg
     P  *  S  V  L  N  L  A  L  S  P  G  W  S  A  V  V  R  Y  W
 363 ctcactgcaacctccgcctctcgggttcaagcgattatcctgcctcagccttttgagtag
     L  T  A  T  S  A  S  R  V  Q  A  I  I  L  P  Q  P  F  E  *
 423 ctgggactacaggcacatggtaccatgccagactaattttttgtattttttagtagagatgg
     L  G  L  Q  A  H  G  T  M  P  D  *  F  L  Y  F  *  *  R  W
 483 gattatcactatgttggccaagatggttttgatctcttgacttcgtgatccgccctcctc
     D  Y  H  Y  V  G  Q  D  G  F  D  L  L  T  S  *  S  A  L  L
 543 ggcctcccaaagtgctgggatttcaggcgttagccaccgcacccagcctggacttgtcta
     G  L  P  K  C  W  D  F  R  R  *  P  P  H  P  A  W  T  C  L
 603 tttcatttggagaacttcaattgatctaaatctactttgcagcttttcattgttaatttt
     F  H  L  E  N  F  N  *  S  K  S  T  L  Q  L  F  I  V  N  F
 663 gaatttatttaaacaagtaaaatatttaagacatttgtgtggaaaacctgtaacaaattg
     E  F  I  *  T  S  K  I  F  K  T  F  V  W  K  T  C  N  K  L
 723 catttggttcaatccctgattatgttttagatatgccaactctttttctctcttttttta
     H  L  V  Q  S  L  I  M  F  *  I  C  Q  L  F  F  S  L  F  L
 783 aaaatttgataaatactgaaaccaattcaatttagatcaaatacattacatttccactag
     K  I  *  *  I  L  K  P  I  Q  F  R  S  N  T  L  H  F  H  *
 843 agggaggaaaagcgctaatttttgaacctttatgagggaactgtgcttttgccgagataat
     R  E  E  K  R  *  F  *  T  F  M  R  E  L  C  F  C  R  D  N
 903 cacttccctcattacacacaaactgtgtctggaaaaacaacagtagttcctttcattttt
     H  F  P  H  Y  T  Q  T  V  S  G  K  T  T  V  V  P  F  I  F
 963 atttaaatctcttctctaatgggaaaacaaaggacaaaacagggaacacttctaggtcaa
     I  *  I  S  S  L  M  G  K  Q  R  T  K  Q  G  T  L  L  G  Q
1023 agtagcaaatctttaaaagtggctaatttcttgcttcaagaattctaaatacagcccccca
     S  S  K  S  L  K  V  A  N  F  L  L  Q  E  F  *  I  Q  P  P
1083 gggtcagaaaaatattc 1099
     G  S  E  K  Y
```

Figure S3. Characterization of lncRNA-CMPK2. (continued). (C, D and E) Analysis of the predicted ORFs in the major isoform of lncRNA-CMPK2 in the three reading frames as determined by the NCBI ORF finder software. For each reading frame, the top panel displays locations of the potential start (turquoise) and stop (magenta) codons. The bottom panel displays nucleotide and deduced amino acid sequences of lncRNA-CMPK2. The potential start (turquoise) and stop (magenta) codons are corresponding to the top panel. The longest predicted ORF in panel D is marked by asterisks on top.

Figure S3F: Analysis of the predicted ORFs for protein-coding capacity

```
1) Longest predicted ORF in human lncRNA-CMPK2

Human           1 tcaaaaggctgaggcaggataatcgcttgaacccgagaggcggaggttgcagtgagccaatatcgcaccactgcactccagcctggcgacagagcaa  97
Chimp             .................................t...............................................................
Gorilla           ......................................................................................t..........
Orangutan         .......................t.................................tg......................................
Gibbon            .......................t...................c..............g............................a.........
Rhesus            .......a...........g....t......t...g..............------------------------------------------.....
Macaque           .......a...........g....t......t...g..............------------------------------------------.....
Baboon            .......a...........g....t......t...g..............------------------------------------------.....
Syn/nonsyn               N           N   NN      N S N       N             NN                           NS

Human          98 gattcagtacagattaaggaggttagaggtgtctgctgcaataatccagaggaagtcttcactggatgagacagaatgctgtacacaaagtgtttagtcttcccatgcat 207
Chimp             .................a............................................................................................
Gorilla           .................-.....................................g......................................................
Orangutan         t................-......g............t............c....a.......a................a.g...........................
Gibbon            .....g...........-....c.g..........t..............c....g..a.......................g.........................t.
Rhesus            ....tg........c..-......g.........................a....g..........................g........a...c..............
Macaque           ....tg........c..--.....g......c..................a....g..........................g........a...c..............
Baboon            ....tg........c..-......g.........................a....g..........................g........a..................
Syn/nonsyn        N   NS        S  S    N N      N   S N            S    N  N    N                S N        N   S

Dn/Ds=21/9 (includes one nonsense codon). In addition, there is a frame shift-inducing indel in all non-human primates except chimp.


2) Predicted ORF located at nts 981-1070 in human lncRNA-CMPK2

Homo sap ATGGGAAAACAAAGGACAAAACAGGGAACACTTCTAGGTCAAAGTAGCAAATCTTTAAAAGTGGCTAATTTCTTGCTTCAAGAATTCTAA
Homo sap  M  G  K  Q  R  T  K  Q  G  T  L  L  G  Q  S  S  K  S  L  K  V  A  N  F  L  L  Q  E  F  *
          M  G  K  Q  R  T  -  -  -  T  -  L  G  Q  S  S  K  S  -  K  V  A  N  F  -  L  Q  E  F  *
Mus musc  M  G  K  Q  R  T  D  *  R  T  F  L  G  Q  S  S  K  S  F  K  V  A  N  F  V  L  Q  E  F  *
Mus musc .....G............G.CTGAA.....T...................G.....C...............G.................

Dn/Ds=6/2


3) Predicted ORF located at nts 873-968 in human lncRNA-CMPK2

Homo sap        ATGAGG-GAACTGTGCT-TTTGCCGAGATAATCACTTCCCTCATTACACACAAACTGT---GTCTGGAAAAACAACAGTAGTTCCTTTCATTTTTATTTAA
Homo sap         M  R   E  L  C   F  C  R  D  N  H  F  P  H  Y  T  Q  T  V  -  S  G  K  T  T  V  V  P  F  I  F  I  *
                 -  -   E  L  -  -  -   -  D  N  H  F  -  H  -  -  Q  T  -  -  -  G  K  T  T  V  V  P  F  I  F  I  *
Mus musc          P  G  E  L  L  C  L   L  D  N  H  F  S  H  *  A  Q  T  A  P  P  G  K  T  T  V  V  P  F  I  F  I  *
Mus musc        -CC...A..G...CT..G.....-TT.....C.....TT.......AG.........CACCGC......................................
Dn/Ds=11/3
```

Figure S3. Characterization of lncRNA-CMPK2. (continued). (F) Analysis of the protein-coding capacity of the short predicted ORFs in lncRNA-CMPK2. Part 1: The longest predicted ORF (69 aa long) was located in a region of the RNA that was not conserved in non-primates. Analysis of its conservation pattern among primates indicated the presence of sequence variations in the start codon in addition to frameshift-inducing indels (both highlighted in red). The shown sequence is in the antisense orientation compared to the direction of transcription of lncRNA-CMPK2. The human sequence is shown on top. The dots indicate positions where the sequence is identical to that in human. The sequence variations are shown. N: non-synonymous. S: synonymous sequence variation. In the red highlighted region, the sequence variation to "a" results in a synonymous amino acid change. Parts 2 and 3: Analysis of two short predicted ORFs that were in a region of the lncRNA that showed conservation among mammalians. The sequence of the ORF (in sense orientation relative to the direction of transcription of lncRNA CMPK2) is shown for human and mouse, with the translation into amino acids shown in between. The middle line in each case contains the result of comparison between the two translated sequences. Minus signs indicate a non-synonymous change. * denotes the position of stop codons. In all three parts, the non-synonymous to synonymous ratio (Dn/Ds) is shown.

Figure S3 (continued)

G

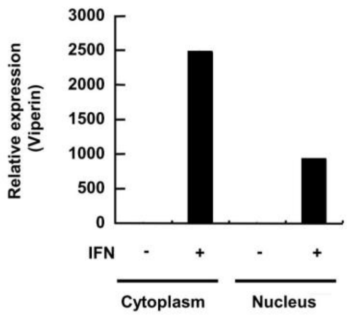

Figure S3. Characterization of lncRNA-CMPK2. (continued). (G) RT-qPCR analysis of the nuclear and cytoplasmic fractions from control and IFN-stimulated Huh7.5 cells for the expression of the ISG viperin. Plus and minus signs indicate the presence and absence of IFN stimulation.

Figure S4

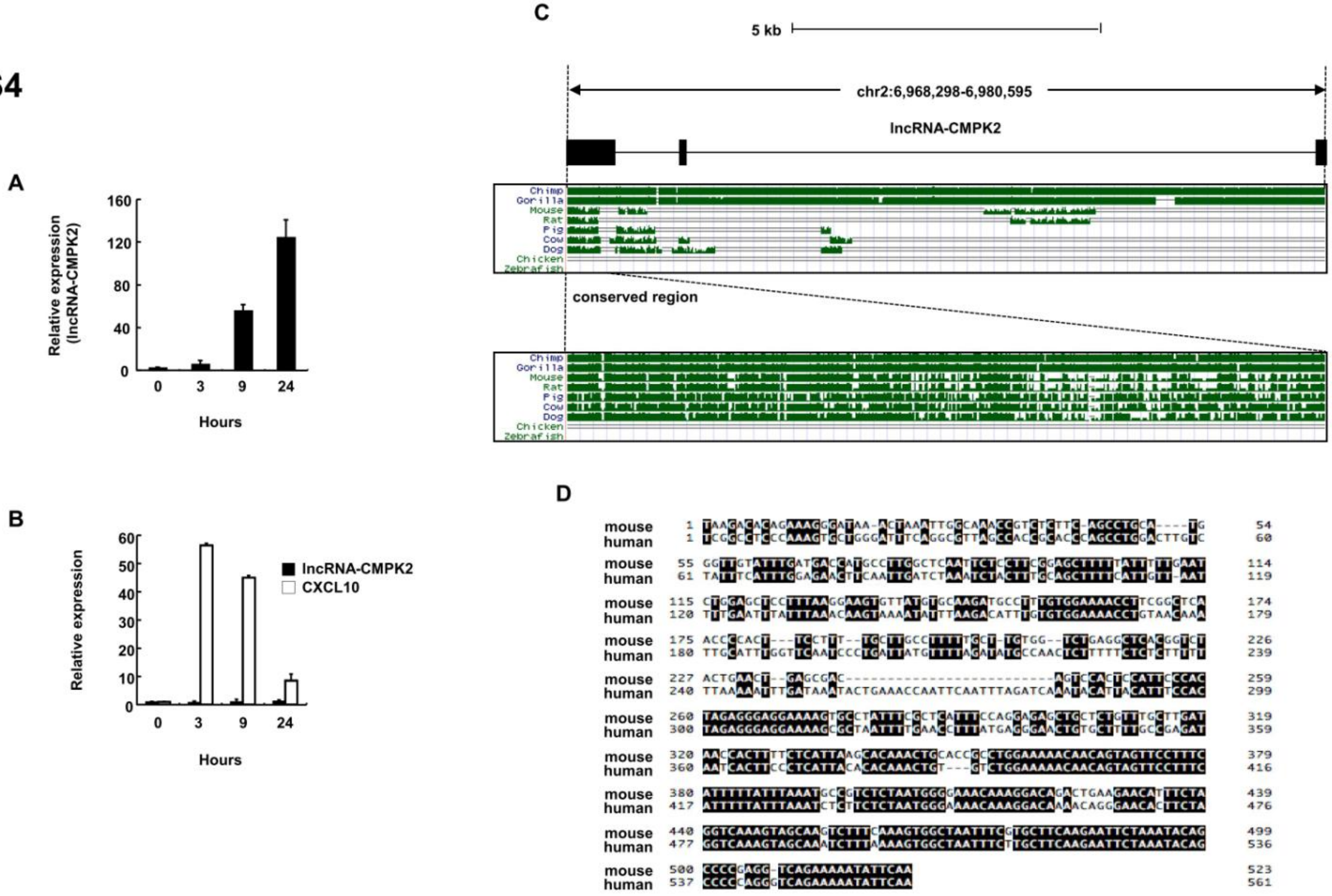

Figure S4. Characterization of expression pattern and conservation of lncRNA-CMPK2. (A) lncRNA-CMPK2 is induced in response to stimulation with the type II interferon, IFN-γ, in Huh7.5 cells. The time points after IFN-γ treatment at which the cellular RNA was harvested is shown at the bottom. (B) lncRNA-CMPK2 is not induced in response to TNF-α stimulation. The numbers at the bottom of the panel indicate the time points after TNF-α stimulation. The expression level of CXCL10, which is induced in response to both IFN and TNF-α, is shown as positive control. (C) Multi-species alignment of the lncRNA-CMPK2 genomic region from the USCS genome browser (hg19). The region that is conserved among mammals is expanded in (D), which shows the alignment of the conserved region of lncRNA-CMPK2 between mouse and human at nucleotide level.

Fig. S5. The values presented in Fig. 4C prior to normalization of the values from IFN(+) samples to the value in the IFN(+) shControl datapoint.

	Average	Standard error	Average	Standard error
Relative HCV RNA	IFN (-)	IFN (-)	IFN (+)	IFN (+)
shlncRNA-CMPK2 #1	1.22	0.066	0.117	0.005
shlncRNA-CMPK2 #2	1.36	0.291	0.088	0.005
shControl	1	0.131	0.3	0.036
