# Supplementary material for: Negative regulation of the interferon response by an interferon-induced long non-coding RNA
Source: Nucleic Acids Res. 2014 Aug 13;42(16):10668–80. doi: 10.1093/nar/gku713 (PMC4176326; doi:10.1093/nar/gku713)
Supplement: SUPPLEMENTARY DATA [file supp_gku713_nar-01057-r-2014-File010.docx]

**Table S3. Experimental and biographical information related to human liver samples. The presence and absence of the HCV genomic RNA was verified by RT-qPCR.** ND: not detectable.

| Subject | Age | Gender | lncRNA-CMPK2 relative expression level (corresponding to Fig. 5C) | HCV  clinically verified | HCV experimentally verified (by RT-PCR) |
| --- | --- | --- | --- | --- | --- |
| 1 | 48 | M | ND | positive* | negative |
| 2 | 60 | M | ND | negative | negative |
| 3 | 44 | F | 0.047321588 | negative | negative |
| 4 | 64 | F | 0.025035474 | negative | negative |
| 5 | 66 | M | ND | negative | negative |
| 6 | 53 | M | 0.016061192 | positive | positive |
| 7 | 63 | F | 0.037016916 | positive | positive |
| 8 | 52 | M | 0.4423219 | positive | positive |
| 9 | 44 | M | 0.056969168 | positive | positive |
| 10 | 42 | M | 3.566825931 | positive | positive |
| 11 | 52 | F | 0.959264119 | positive | positive |

*HCV antibody positive, prior successful treatment resulting in sustained virologic response.
